# Down-regulation of the Antisense Mitochondrial Non-coding RNAs (ncRNAs) Is a Unique Vulnerability of Cancer Cells and a Potential Target for Cancer Therapy*

**DOI:** 10.1074/jbc.M114.558841

**Published:** 2014-08-06

**Authors:** Soledad Vidaurre, Christopher Fitzpatrick, Verónica A. Burzio, Macarena Briones, Claudio Villota, Jaime Villegas, Javiera Echenique, Luciana Oliveira-Cruz, Mariela Araya, Vincenzo Borgna, Teresa Socías, Constanza Lopez, Rodolfo Avila, Luis O. Burzio

**Affiliations:** From the ‡ Andes Biotechnologies SA and; ¶ Fundación Ciencia para la Vida, Zañartu 1482, Ñuñoa, Santiago 7780272, Chile,; ‖ Facultad de Ciencias Biológicas and; ** Facultad de Medicina, Universidad Andrés Bello, República 252, Santiago 8370134, Chile, and; § Departamento de Ciencias Químicas y Biológicas, Universidad Bernardo ÓHiggins, General Gana 1702, Santiago, Chile

**Keywords:** Apoptosis, Cancer, MicroRNA (miRNA), Mitochondria, Survivin, ncRNA

## Abstract

Hallmarks of cancer are fundamental principles involved in cancer progression. We propose an additional generalized hallmark of malignant transformation corresponding to the differential expression of a family of mitochondrial ncRNAs (ncmtRNAs) that comprises sense and antisense members, all of which contain stem-loop structures. Normal proliferating cells express sense (SncmtRNA) and antisense (ASncmtRNA) transcripts. In contrast, the ASncmtRNAs are down-regulated in tumor cells regardless of tissue of origin. Here we show that knockdown of the low copy number of the ASncmtRNAs in several tumor cell lines induces cell death by apoptosis without affecting the viability of normal cells. In addition, knockdown of ASncmtRNAs potentiates apoptotic cell death by inhibiting survivin expression, a member of the inhibitor of apoptosis (IAP) family. Down-regulation of survivin is at the translational level and is probably mediated by microRNAs generated by dicing of the double-stranded stem of the ASncmtRNAs, as suggested by evidence presented here, in which the ASncmtRNAs are bound to Dicer and knockdown of the ASncmtRNAs reduces reporter luciferase activity in a vector carrying the 3′-UTR of survivin mRNA. Taken together, down-regulation of the ASncmtRNAs constitutes a vulnerability or Achilles' heel of cancer cells, suggesting that the ASncmtRNAs are promising targets for cancer therapy.

## Teaser

**Background:** Down-regulation of the antisense mitochondrial ncRNAs (ASncmtRNAs) is a general vulnerability of cancer cells.

**Results:** Knocking down the ASncmtRNAs induces apoptosis, down-regulation of survivin, and generation of microRNAs.

**Conclusion:** Induction of apoptosis in cancer cells is potentiated by down-regulation of survivin.

**Significance:** The ASncmtRNAs represent a new potential therapeutic target for cancer.

## Introduction

An inspiring compilation of human cancer hallmarks was described by Hanahan and Weinberg (1). These hallmarks include sustained proliferation, immortality, refractoriness to growth suppressors, resistance to cell death, angiogenic capability, and induction of invasion and metastasis promoted by genome instability, mutation, and inflammation (1). Energy metabolism reprogramming (2), evasion of the immune system (3), and the tumor microenvironment (4) are additional factors influencing cancer progression.

We reported the differential expression in human cells of a unique family of mitochondrial long ncRNAs^4^ (ncmtRNAs) containing stem-loop structures (5, 6). Interestingly, these transcripts exit mitochondria to the cytosol and the nucleus, suggesting a functional role for these molecules outside the mitochondria (7). One of these transcripts, sense ncmtRNA (SncmtRNA), is expressed in normal proliferating cells and tumor cells but not in resting cells, suggesting a role for this molecule in cell cycle progression (5, 6, 8). In addition to SncmtRNA, normal human proliferating cells express two antisense mitochondrial ncRNAs (ASncmtRNA-1 and ASncmtRNA-2) (6). Remarkably, however, the ASncmtRNAs are down-regulated in multiple tumor cell lines and tumor cells present in 17 types of cancer biopsies from different patients (6). A hallmark of carcinogenesis is down-regulation of tumor suppressors involving several mechanisms (9). Therefore, we postulated that the ASncmtRNAs might function as a unique mitochondria-encoded tumor suppressor (6). To test this possibility, we investigated the expression of the ASncmtRNAs in cell lines immortalized with oncogenic high risk human papilloma virus (HPV 16 or 18). Immortalization of keratinocytes with these viruses induces down-regulation of two important tumor suppressors: p53 and Rb (10). Similarly, we observed down-regulation of ASncmtRNAs in keratinocytes immortalized with the complete genome of HPV 16 and 18 (8). Moreover, we show that down-regulation of these transcripts requires expression of the viral E2 oncogene (8), which allows the theory that in non-viral-induced cancers down-regulation of the ASncmtRNAs could be triggered by as yet unknown cellular oncogenes. Hence, down-regulation of the ASncmtRNAs seems to be an important step in carcinogenesis and represents a new hallmark of cancer (1).

Hypothetically, down-regulation of the ASncmtRNAs may also represent a general vulnerability of cancer cells (11–13). Therefore, we asked whether ASncmtRNA knockdown (ASK for short) induces alteration of cancer cell function. Here we show that ASK on HeLa cells with antisense oligonucleotides (ASOs) directed to the ASncmtRNAs induces strong inhibition on proliferation. Surprisingly, and in addition to decreased proliferation, ASK induced caspase-dependent apoptosis (14) of HeLa cells and several other tumor cell lines without affecting viability of normal cells. In addition and potentiating the onset of apoptotic cell death, ASK induces a drastic reduction in the levels of survivin, a member of the inhibitor of apoptosis (IAP) family, which is up-regulated in virtually all human cancers (15–18). Notably also, anchorage-independent growth (19) is markedly reduced by ASK. Furthermore, we determined that down-regulation of survivin induced by ASK occurs at the translational level and may be mediated by microRNAs (miRs) (20–24). Supporting this idea, we demonstrate that ASncmtRNAs are co-immunoprecipitated with Dicer in whole-cell lysates. Furthermore, by using a luciferase reporter assay (25, 26), we demonstrate that ASK induces reduced luciferase activity in a construct containing the 3′-UTR of survivin mRNA downstream of the firefly luciferase gene. To our knowledge this work represents the first report on the identification of mitochondrial ncRNAs as potential targets for cancer therapy and on the generation of putative microRNAs (Mito-miRs?) probably from the stem of these transcripts.

## EXPERIMENTAL PROCEDURES

### 

#### 

##### Cell Culture

Pooled neonatal human foreskin keratinocytes (HFK) were purchased from Lonza (Basel, Switzerland) and cultured in keratinocyte serum-Free medium (Invitrogen). All remaining human tumor cell lines and normal primary cells were obtained from ATCC. HeLa and SiHa (cervix carcinoma, transformed with HPV 18 and 16, respectively), MDA-MB-231 and MCF7 (breast carcinoma), DU-145 and PC-3 (prostate carcinoma), HepG2 (hepatoma), OVCAR-3 (ovarian carcinoma), Caco-2 (colon carcinoma), U87 (glioblastoma), SK-MEL-2 (melanoma), A498 (renal carcinoma), H292 (lung carcinoma), human normal epidermal melanocytes (HnEM), and human normal renal epithelial cells (HREC) were cultured according to ATCC guidelines. Human umbilical vein endothelial cells (HUVEC) were cultured in M199 media (Invitrogen) supplemented with 20% FCS, 50 μg/ml heparin, 50 μg/ml endothelial cell growth supplement (Calbiochem), and penicillin/streptomycin (50 units/ml penicillin, 50 μg/ml streptomycin). All cell cultures were maintained in a humidified cell culture chamber at 37 °C and 5% CO_2_. Cell cultures were checked periodically for mycoplasma contamination using the EZ-PCR Mycoplasma Test kit (Biological Industries).

##### Fluorescence in Situ hybridization (FISH)

FISH, for detection of SncmtRNA and ASncmtRNAs, was performed on monolayer cultures on glass slides (Slide Chambers, Nunc) according to the protocol described before (6).

##### Cell Transfection

Antisense oligonucleotides (ASOs) used in this study were synthesized by Integrated DNA Technologies or Invitrogen with 100% phosphorothioate (PS) linkages. ASOs with peptidic linkages (PNA) were synthesized by PNA Bio (Thousand Oaks, CA). The sequences of the ASOs utilized were 5′-CACCCACCCAAGAACAGG (ASO-1537S), 5′-GTCCTAAACTACCAAACC (ASO-1107S), 5′-TACCTAAAAAATCCCAAACA (ASO-552S), 5′-AGATGAAAAATTATAACCAA (ASO-126S), and 5′-AGGTGGAGTGGATTGGGG-3′ (control ASO (ASO-C)). Some ASOs were labeled at the 5′ end with Alexa Fluor 488 in order to assess transfection efficiency. For ASO treatments, cells were seeded into 12-well plates (Nunc) at 25,000–50,000 cells/well and transfected the next day with ASOs at concentrations ranging from 50 to 200 nm using Lipofectamine2000 (Invitrogen), according to the manufacturer's directions. Transfection was allowed to proceed for 18–48 h under normal culture conditions. The amount of ASO and Lipofectamine2000 for each cell line is listed in Table 1. Peptidic nucleic acid (PNA) oligonucleotides were transfected under the same conditions.

##### Proliferation and Cell Viability

Total cell number and viability was determined by trypan blue (Tb) exclusion or propidium iodide (PI). PI was added at 1 μg/ml 1 min before flow cytometry on a BD Biosciences FACS Canto Flow Cytometer (Universidad de Chile). For Tb, the number of viable cells was determined counting at least 100 cells per sample in triplicate. Cell proliferation rate was measured with the Click-iT® EdU Alexa Fluor® 488 kit (Invitrogen) according to manufacturer's directions. Samples were analyzed on an Olympus BX-51 fluorescence microscope.

##### Conventional and Quantitative RT-PCR Amplification

Unless otherwise specified, RNA was extracted with TRIzol reagent (Invitrogen) as described (5, 6, 8). To eliminate genomic and mitochondrial DNA contamination, RNA preparations were treated with TURBO DNA-free (Ambion) according to manufacturer's instructions. For conventional RT-PCR, reverse transcription was carried out with 50–100 ng of RNA, 50 ng of random hexamers, 0.5 mm each dNTP, 5 mm DTT, 2 units/μl RNase-out (Invitrogen), and 200 units of reverse 25 transcriptase (Moloney murine leukemia virus; Invitrogen). Reactions were incubated at 25 °C for 10 min, 37 °C for 50 min, and 65 °C for 10 min. PCR was carried out in 50 μl containing 2 μl of cDNA, 0.4 mm each dNTP, 1.5 mm MgCl_2_, 2 units GoTaq (Promega), and 1 μm each forward (for) and reverse (rev) primer (18 S: for, 5′-AGTGGACTCATTCCAATTA; rev, 5′-GATGCGTGCATTTAT; ASncmtRNA-1: for, 5′-TAGGGATAACAGCGCAATCCTATT; rev, 5′-CACACCCACCCAAGAACAGGGAGGA; ASncmtRNA-2: for, 5′-ACCGTGCAAAGGTAGCATAATCA; rev, 5′-ACCCACCCAAGAACAGG) in the appropriate buffer. The amplification protocol consisted of 5 min at 94 °C, 30 cycles of 94 °C, 58 °C, and 72 °C for 1 min each, and finally, 72 °C for 10 min, with the exception of 18 S amplification reactions, which consisted of only 15 cycles. For quantitative RT-PCR, cDNA was synthesized with the Affinity Script QPCR cDNA Synthesis kit (Agilent Technologies) using 500 ng of RNA and 250 ng of random hexamers (Invitrogen). Reactions were incubated for 10 min at 25 °C, 1 h at 45 °C, and 5 min at 95 °C. RNase H (2 units) was added, and samples were incubated at 37 °C for 20 min. Real-time PCR (quantitative PCR) for survivin was carried out on a Stratagene Mx3000PTM Real-time PCR System (Agilent Technologies) with 3 μl of a 1:5 cDNA dilution, 1× GoTaq Flexi Buffer, 2 mm MgCl_2_, 0.4 mm each dNTP, 2.5 units of GoTaq DNA polymerase, 0.5 μm each forward and reverse primer, and 0.25 μm Taqman probe (Survivin: forward, 5′-ATGGGTGCCCCGACGT; reverse, 5′-AATGTAGAGATGCGGTGGTCCTT; probe, 5′-CCCCTGCCTGGCAGCCCTTTC) and in a volume of 25 μl. Cycle parameters were 95 °C for 2 min and 40 cycles of 95 °C for 15 s, 54 °C for 15 s, and 62 °C for 45 s. Results were normalized to the average of the housekeeping controls RPL27 mRNA (forward, 5′-AATCACCTAATGCCCAC; reverse, 5′-TGTTCTTGCCTGTCTTG; probe, 5′-CAGAGATCCTGCTCTTAAACGC) and 18 S rRNA (forward, 5′-GTAACCCGTTGAACCCCATT; reverse, 5′-CATCCAATCGGTAGTAGCG, probe 5′-AGTAAGTGCGGGTCATAAGCTTGCGT), and data were analyzed by analysis of variance. For end-point RT-PCR amplification of mitochondrial mRNAs of ND1 and COX1 and 12 S rRNA, we used the following primers: ND1 (forward, 5′-CCCTAAAACCCGCCACATCT; reverse, 5′-CGATCAGGGCGTAGTTTGA); COX1 (forward, 5′-TCTCCTACTCCTGCTCGCAT; reverse, 5′-GGGTTATGGCAGGGGGTTTT); 12 S rRNA (forward, 5′-AATAGGTTTGGTCCTAGCCTTTCTATTA; reverse, 5′-ATTGACCAACCCTGGGGTTAGTATA); control, 18 S rRNA (same primers as for quantitative PCR). ND1 and COX1 were amplified with 25 cycles. Amplification of 12 S and 18 S was carried out for 20 and 15 cycles, respectively.

##### Transmission Electron Microscopy (TEM)

HeLa cells were prepared for TEM as described before (7), and Images were acquired on a Philips Tecnai 12 BioTwin transmission electron microscope (Pontificia Universidad Católica de Chile), operating at 80 kV.

##### Measurement of Mitochondrial Depolarization (Δψm)

Cells seeded at 5 × 10^4^ cells/well in 12-well plates were transfected for 24 h as described above. Afterward, cells were loaded with 20 nm tetramethylrhodamine methyl ester (Molecular Probes) for 15 min at 37 °C (27), harvested, and analyzed by flow cytometry on a BD Biosciences FACS Canto Flow Cytometer, using the PE setting. As a positive control, mitochondrial depolarization was elicited using 10 μm carbonyl cyanide 3-chlorophenylhydrazone (CCCP; Sigma) for 30 min at 37 °C.

##### Subcellular Fractionation

Cells (2 × 10^6^) were washed twice with ice-cold PBS, suspended in 500 μl Mito buffer (10 mm HEPES, pH 6.8, 0.6 m mannitol, and 1 mm EDTA) containing 1 mm PMSF and protease inhibitor mixture (Sigma), and incubated for 15 min on ice. Cells were then disrupted by 6 cycles of freezing in liquid nitrogen and thawing at 37 °C. The crude lysate was centrifuged at 1500 × *g* for 10 min at 4 °C to sediment whole cells and nuclei. The supernatant was transferred to ice-cold tubes and centrifuged at 10,000 × *g* for 30 min at 4 °C to sediment mitochondria, and the supernatant was recovered. Protein concentration was quantified with the Bradford microplate system Gen5TM EPOCH (BioTek), and samples were analyzed by Western blot.

##### Preparation of Whole Cell Extracts

Cells transfected for 24 h were harvested, washed in ice-cold PBS, and sedimented at 1000 × *g* for 10 min at RT. Pellets were suspended in radioimmunoprecipitation assay buffer (10 mm Tris-HCl, pH 7.4, 1% sodium deoxycholate, 1% Triton X-100, 0.1% sodium dodecyl sulfate) containing 1 mm PMSF and a protease inhibitor mixture (Sigma). Protein concentration was quantified as described above.

##### Western Blot

Proteins (30 μg/lane) were resolved by SDS-PAGE and transferred to polyvinylidene difluoride membranes. Membranes were probed with antibodies against cytochrome *c* (rabbit polyclonal; Cell Signaling; 1:1000), survivin (rabbit polyclonal; R&D systems; 1:1000), or XIAP (rabbit monoclonal; Cell Signaling: 1:1000) and revealed with peroxidase-labeled anti-mouse or anti-rabbit IgG (Calbiochem; 1:5000). Blots were detected with the EZ-ECL system (Biological Industries). Mouse monoclonal anti-β-actin (Sigma; 1:4000) or anti-GAPDH (mouse monoclonal; Abcam: 1:2000) were used as a loading control. The pixel intensity of each protein band was quantified using ImageJ software (National Institutes of Health).

##### DNA Fragmentation

DNA fragmentation was evaluated by the DeadEnd^TM^ Fluorometric TUNEL kit (Promega) according to the manufacturer's directions and flow cytometric quantification of hypodiploid cells (sub-G_1_ fraction). For quantification of hypodiploid events, 10^5^ cells/well were transfected for 48 h as described above. Staurosporine (STP, Sigma) was used at a concentration of 5 μm as a positive control of apoptosis. Cells were harvested and centrifuged at 600 × *g* for 5 min. Pellets were suspended in 100% ethanol and stored at −20 °C for 24 h. Cells were then treated with 1 mg/ml RNase A for 1 h at RT. PI was added, and samples were subjected to flow cytometry.

##### Determination of Phosphatidylserine Exposure

Phosphatidylserine exposure was determined by annexin-V binding with the APOtarget kit (Invitrogen) according to manufacturer's directions and analyzed by flow cytometry or fluorescence microscopy.

##### Caspase Activation

Caspase activation was determined using the fluorogenic caspase inhibitor CaspACE^TM^ FITC-VAD-fmk (Promega). After transfection, FITC-VAD-fmk was added at 10 μm and incubated 20 min at 37 °C. Cells were harvested, washed in PBS, and fixed in 3.7% *p*-formaldehyde for 15 min at RT. Fluorescent images were obtained as above. Inhibition of apoptosis in treated cells was carried out with the non-fluorescent caspase inhibitor z-VAD-fmk (Promega) added 2 h before transfection at a concentration of 25 μm (HeLa) or 40 μm (SK-MEL-2) for 24 h, and cell death was analyzed by Tb exclusion.

##### Colony Formation Assay

Anchorage-independent cell growth was determined by colony formation in soft agar as described (19). Treated cells were seeded at 200–2000 live cells/well in 12-well plates in soft agar. Formation of colonies >50 μm in diameter was monitored at 2–3 weeks.

##### RNA Immunoprecipitation

RNA immunoprecipitation experiments were carried out with the MagnaRIP kit (Merck Millipore). Briefly, 2 × 10^7^ SK-MEL-2 cells were washed in 10 ml of ice-cold sterile PBS, scraped, sedimented at 300 × *g* for 5 min at 4 °C, and lysed in 100 μl of RIP lysis buffer containing 0.5 μl protease inhibitor and 0.25 μl of RNase inhibitor (included in the kit). An aliquot of 10 μl of each lysate was stored at −80 °C (input), and for protein/RNA immunoprecipitation, 100 μl of lysate was mixed with 900 μl of a suspension of magnetic beads previously loaded with 5 μg of anti-Dicer monoclonal antibody (Abcam) or polyclonal anti-SNRNP70 or control mouse or rabbit IgG (supplied with the kit) at RT for 30 min under rotation. The cell lysate/magnetic beads/antibody mix was incubated at 4 °C for 4 h under rotation. After magnetic separation of RNA-protein complexes, a wash was performed in 500 μl of immunoprecipitation wash buffer followed by 5 washes in RIP wash buffer. For RNA purification, immunoprecipitates (and inputs) were incubated at 55 °C for 30 min in 150 μl of proteinase K buffer (RIP wash buffer containing 1% SDS and 1.8 mg/ml proteinase K) under constant agitation. After magnetic bead separation, supernatants were transferred to different tubes, and 250 μl of RIP wash buffer was added followed by 400 μl of phenol:chloroform:isoamyl alcohol (125:24:1, pH 4.5). After mixing, phase separation was performed at 21,000 × *g* for 10 min at RT. The aqueous layer was mixed with 40 ml of chloroform and centrifuged at 21,000 × *g* for 10 min at RT. The aqueous phase was recovered, and 300 μl was mixed with 50 μl of salt solution I, 15 μl of salt solution II, 5 μl of precipitate enhancer (supplied in the kit), and 850 μl of ethanol. RNA was precipitated overnight at 4 °C. RNA was recovered by centrifugation at 21,000 × *g* for 30 min at 4 °C, the pellet was washed twice in ice-cold 70% ethanol followed by centrifugation at 21,000 × *g* for 15 min at 4 °C, and the RNA pellet was resuspended in nuclease-free water. Synthesis of cDNA and PCR were performed as described above utilizing 60 ng of RNA from each sample using primer ASrev (5′-ACCCACCCAAGAACAGG) with AS1-for (5′-TAGGGATAACAGCGCAATCCTATT) for amplification of ASncmtRNA-1 and with AS2-for (5′-GAACTCGGCAAACCTTACC) for amplification of ASncmtRNA-2. Control RNA (U1 snRNA) was amplified using primers supplied in the kit.

##### Bioinformatics

The double-stranded region of the ASncmtRNA-2 (GenBank^TM^ accession number EU863790) was processed *in silico* using either the 3′ counting or the 5′ counting method (28, 29) to generate fragments of 19 to 25 bp. Then the presence of a “seed” sequence of 7–8 nucleotides at the 5′ end of each fragment was established by blastn alignment (blast.ncbi.nlm.nih.gov), TargetScan18 or miRBase (28, 29) and the 3′-UTR of survivin (BIRC5) mRNA (GenBank^TM^ NM_001168). The seed sequence was compared with miRs described for survivin for similar thermodynamic stability and without considering G:U wobble.

##### Luciferase Assay

The 3′-UTR of the survivin mRNA was amplified by RT-PCR from total RNA of SK-MEL-2 cells using forward primer 5′-AAAAAATCTAGACTTGTTTTGTCTTGAAAGTGGCACCAG and reverse primer 5′-AAAAAATCTAGAGCACCACTTCCAGGGTTTATTCC and cloned into a unique XbaI site downstream of the firefly luciferase ORF in the pmiRGLO dual-luciferase vector (Promega). The identity of the 3′-UTR region was verified by DNA sequencing, and only those clones in the 5′ to 3′ orientation with respect to the firefly luciferase gene were used. SK-MEL-2 cells were plated into 12-well plates (Nunc) at a density of 50,000 cells/well. On the next day cells were transfected with pmiRGLO empty vector or containing the survivin 3′-UTR at 0.5 μg/well using Lipofectamine2000 (Invitrogen) and, 24 h later, with 150 nm control ASO (ASO-C) or ASO 1537S, also using Lipofectamine2000. A positive control with 20 nm survivin siRNA (5′-AGACAGAATAGAGTGATAGGAAGCG and 5′-CGCUUCCUAUCUCUAUUCUGUCU) targeted to positions 1263–1287 of the 3′-UTR of survivin mRNA (Integrated DNA Technologies) (access number NM_001168, mRNA variant1 BIRC5) was included. At 24 h post-transfection of ASOs, cells were detached by trypsinization, sedimented at 300 × *g* for 5 min at RT, and left in a final volume of 100 μl. Seventy-five μl of each cell suspension was deposited into a well of a 96-well white plate for luminescence, and firefly and Renilla luciferase activity were determined sequentially and immediately on a Synergy H4 Plate reader (BioTek) using the Dual-Glo Luciferase Assay System (Promega) according to manufacturer's directions. Values are expressed as firefly/Renilla relative luciferase activity normalized to the determinations obtained with the empty vector.

##### Statistical Analysis

Experiments were performed at least in triplicate. Results were analyzed by Student's *t* test and represent the average ± S.E. Significance (*p* value) was set at the nominal level of *p* < 0.05 or less.

## RESULTS

### 

#### 

##### Knockdown of ASncmtRNAs Induces Decreased Proliferation of HeLa Cells

**FIGURE 1.**
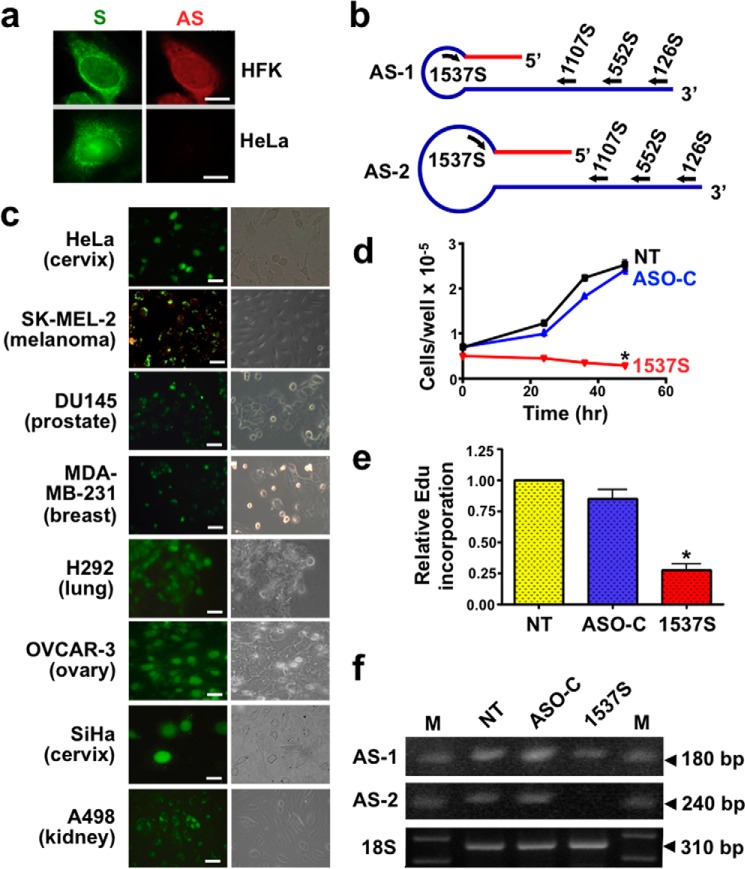
**ASK induces inhibition of proliferation in HeLa cells.** *a*, differential expression of the SncmtRNA (*S; green*) and ASncmtRNAs (*AS; red*) in human keratinocytes and HeLa cells as observed by FISH. *b*, schematic representation of the AS-1 and -2, indicating the relative position of the ASOs utilized in this work. *c*, transfection of different tumor cell lines with fluorescently labeled ASO-1537S. The indicated cell lines were seeded at 5 × 10^4^ cells/well into 12-well plates and transfected the next day with ASO-1537S coupled to Alexa Fluor 488 (see Table 1). At 24 h post-transfection >90% of cells were fluorescently labeled (*left panel*, fluorescence; *right panel*, phase contrast. *Bars* = 20 μm). *d*, proliferation of HeLa cells. Cells (5 × 10^4^ cells/well) were seeded into 12-well plates and transfected the next day with ASO-C or ASO-1537S (see “Experimental Procedures”) or left untreated (*NT*). At the indicated times cells were harvested and counted. At 48 h post-transfection in triplicate, ASK with ASO-1537S induces significant inhibition of cell proliferation as compared with controls (data are presented as the mean ± S.E.; *p < 0.01). *e*, ASK induces inhibition of DNA synthesis. HeLa cells were plated and treated in triplicate as described in *d* for 48 h. The rate of DNA synthesis was measured using the Click-iT® EdU Alexa Fluor® 488 kit, after a 2-h EdU pulse (see “Experimental Procedures”). The cells were co-stained with DAPI and analyzed by fluorescence microscopy. In each experiment the number of EdU-positive cells *versus* the total amount of cells (DAPI staining) was counted in quadruplicate. EdU incorporation was significantly decreased upon transfection with ASO-1537S compared with controls (*, *p* < 0.01). *f*, knockdown of the ASncmtRNAs. After transfection, as described in *d*, HeLa cells were harvested, total RNA was purified, and the expression of the transcripts was determined by RT-PCR. A representative result shows that ASO-1537S induces reduction in the levels of AS-1 and AS-2, compared with controls (*NT* and *ASO-C*). The 18 S rRNA was used as loading control. The size of each amplicon is indicated. *M*, 100-bp ladder.

Fig. 1*a* illustrates the differential expression of the ASncmtRNAs by FISH (6) between normal proliferating cells and tumor cells. HFK express the SncmtRNA and the ASncmtRNAs, whereas in HeLa cells the ASncmtRNAs are down-regulated. Although the ASncmtRNA FISH signal is weak in tumor cells, RT-PCR readily detects a low amount of these transcripts (6, 8). Therefore, we asked whether knocking down this low amount of ASncmtRNAs in tumor cells would induce cellular changes. Fig. 1*b* shows a schematic structure of the ASncmtRNA-1 (AS-1) and ASncmtRNA-2 (AS-2) and the relative position of the ASOs used in this study to induce ASK (see “Experimental Procedures”). The ASOs utilized in this work were PSs, and Lipofectamine2000 (Invitrogen) was used as the transfection agent (see “Experimental Procedures”) under the conditions specified in Table 1. HeLa cells were transfected with ASO-1537S, complementary to the loop region of the ASncmtRNAs or with ASO-C or left untreated (NT). Lipofection for 24 h with Alexa Fluor 488-coupled ASO-1537S (Molecular Probes) revealed a >90% transfection of HeLa and other tumor cell lines (Fig. 1*c*). ASK induces a marked inhibition of cell proliferation compared with untreated cells (*NT*) or cells transfected with ASO-C (Fig. 1*d*). Similarly, the DNA synthesis rate measured by incorporation of the BrdU analog Edu (Molecular Probes) was also inhibited by ∼75% compared with controls (Fig. 1*e*). Knockdown of ASncmtRNAs with ASO-1537S was confirmed by RT-PCR (Fig. 1*f*).

**TABLE 1:** **Conditions for transfection of ASOs in human tumor and normal cell lines, in 12-well multiplates**

Cell Line	ASO concentration	Lipofectamine
	*nm*	μ*l*
**Tumor**		
HeLa	100	2.5
SiHa	100	2.5
MDA-MB-231	100	2.5
MCF7	100	2.5
DU145	100	2.5
HepG2	150	2.5
U87	150	2.5
SK-MEL-2	150	2.0
OVCAR-3	200	2.0
H292	100	2.5
A498	150	4.0

**Normal**		
HFK	100	1.0
HREC	150	2.0
HnEM	150	4.0*^a^*
HUVEC	100	2.5

*^a^* Lipofectin (Invitrogen) was used in place of Lipofectamine2000.

##### ASK Induces Cell Death of Tumor Cell Lines

**FIGURE 2.**
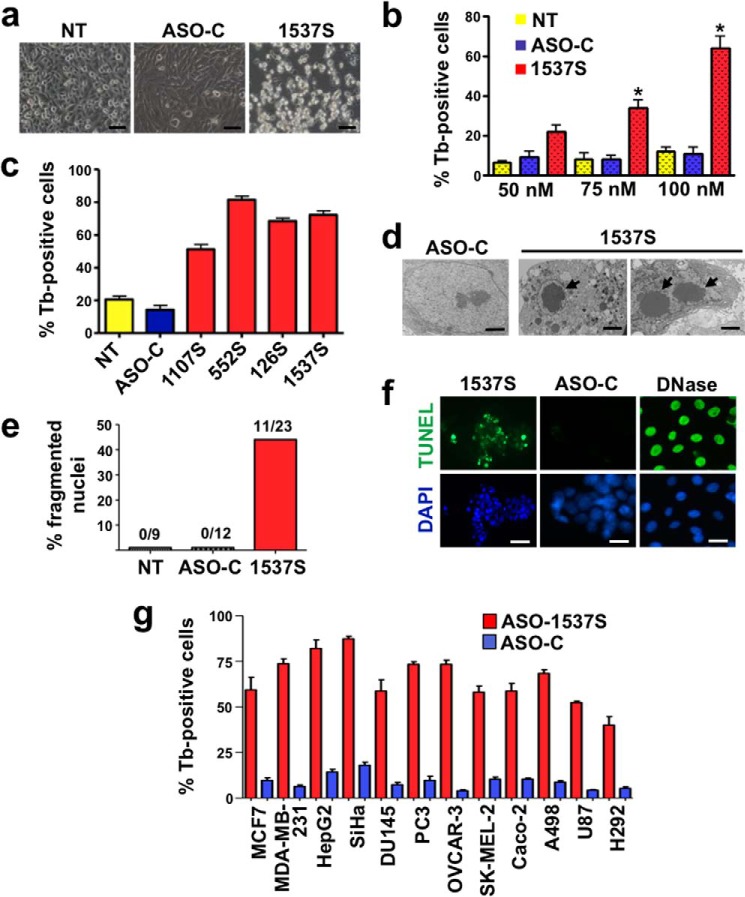
**ASK elicits death of tumor cells.** *a*, ASK induces cell detachment. HeLa cells were seeded at 5 × 10^4^ cells/well in 12-well plates and transfected the next day with 100 nm ASO-C or ASO-1537S or left untreated (*NT*). At 48 h, as observed by phase microscopy, ASO-1537S induced massive cell detachment from the substrate but not in controls (*NT*, *ASO-C*). *Bars* = 50 μm. *b*, effect of ASO concentration on cell death. HeLa cells transfected with the indicated concentrations of ASOs were harvested at 48 h, stained with Tb and counted. Compared with controls (*NT* and *ASO-C*), ASO-1537S induced dose-dependent cell death (%Tb-positive cells), reaching around 70% at 100 nm (*, *p* < 0.01). *c*, effect of different ASOs targeted to the ASncmtRNAs. HeLa cells were transfected in triplicate with 100 nm ASO-C or the indicated ASOs targeted to the 3′ single-stranded region of the ASncmtRNAs (see Fig. 1*b* and “Experimental Procedures”) or left untreated (*NT*). At 48 h post-transfection, specific ASOs induced 50–80% Tb-positive cells. *d*, TEM of HeLa cells transfected with ASO-1537S, but not with ASO-C, for 48 h revealed chromatin condensation (*middle panel*) and nuclear fragmentation (*right panel*) (*arrows*). *Bars* = 2.5 μm. *e*, quantification analysis of TEM microphotographs indicate that ASK induces ∼47% of cells with fragmented nuclei and chromatin condensation. The *numbers on top of each bar* indicate cells with fragmented nuclei and condensed chromatin *versus* total amount of cells analyzed. *f*, ASK causes chromatin fragmentation. HeLa cells transfected with ASO-C or ASO-1537S were harvested at 48 h, fixed, subjected to fluorescent TUNEL assay and counterstained with DAPI (see “Experimental Procedures”). DNase I-treated cells were used as the positive staining control (DNase). ASO-1537S but not ASO-C induced marked DNA fragmentation. *Bars* = 20 μm. *g*, ASK causes cell death in several human tumor cell lines. The indicated cell lines were seeded (5 × 10^4^ cells/well) in 12-well plates. On the next day the cells were transfected for 48 h with ASO-C or ASO-1537S under the conditions listed in Table 1 and analyzed by Tb exclusion. A triplicate analysis shows that ASK induces a significant fraction (50–80%) of Tb-positive cells compared with ASO-C.

Phase contrast microscopy of ASO-1537S-treated HeLa cells (48 h) reveals massive detachment of cells from the substrate, suggesting that ASK causes cell death. In contrast, untreated cells (*NT*) or ASO-C-treated cells showed no significant alteration (Fig. 2*a*). Indeed, a 48-h treatment of HeLa cells with ASO-1537S shows a concentration-dependent increase in the percentage of dead cells as measured by Tb staining (Fig. 2*b*). At 48 h post-transfection with 100 nm ASO-1537S, ∼70% of the cells were trypan blue-positive compared with 8–10% in controls (Fig. 2*b*). Higher concentrations of ASO-1537S (150 or 200 nm) did not reveal major changes. Additional ASOs complementary to other regions of the ASncmtRNAs (see Fig. 1*b* and “Experimental Procedures”) also elicited cell death, at levels similar to ASO-1537S (Fig. 2*c*). TEM of HeLa cells treated for 48 h with 100 nm ASO-1537S revealed chromatin condensation and fragmented nuclei (Fig. 2*d*), suggesting that ASK induces cell death through apoptosis. Quantification analysis of TEM records indicates that ASK induces ∼47% of cells with fragmented nuclei and chromatin condensation (Fig. 2*e*). A telltale feature of apoptosis is chromatin fragmentation. To explore this aspect, HeLa cells treated as described above for 48 h were subjected to fluorescent TUNEL assay using DNase I as the positive control. HeLa cells treated with ASO-1537S were TUNEL-positive compared with ASO-C-treated cells (Fig. 2*f*).

To assess whether ASK induces death in other human tumor cell lines, MCF7 and MDA-MB-231 (breast carcinoma), HepG2 (hepatocarcinoma), SiHa (cervix, HPV 16-transformed), DU145 and PC3 (prostate carcinoma), OVCAR-3 (ovarian carcinoma), SKMEL-2 (melanoma), Caco-2 (colon carcinoma), A498 (renal carcinoma), U87 (glioblastoma), and H292 (lung carcinoma) cells were transfected for 48 h with ASO-1537S or ASO-C (Table 1), and cell death was determined by TUNEL assay. Depending on the cell line, TUNEL-positive cells induced by ASK varied between 50 and 80% (Fig. 2*g*). No significant degree of cell death was observed in the same cells transfected with ASO-C (Fig. 2*g*).

**FIGURE 3.**
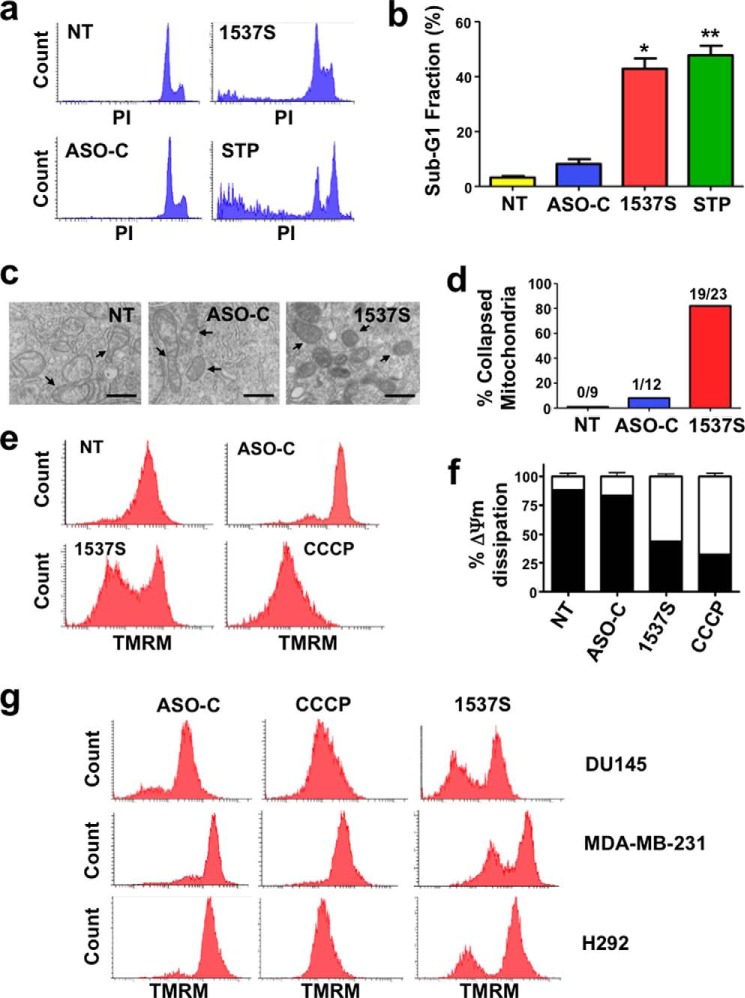
**Mitochondrial and nuclear alterations elicited by ASK.** *a*, ASK induces the increase in sub-G_1_ fraction. HeLa cells treated with ASO-C or ASO-1537S and with STP or left untreated (*NT*) were harvested at 24 h, stained with PI, and analyzed by flow cytometry. Only ASO-1537S or STP elicited an increase in sub-G_1_ DNA fraction. *PI*, PI fluorescence intensity. *b*, compared with controls (NT and ASO-C), a triplicate analysis revealed that ASK induces a significant sub-G_1_ fraction (>40%) (*, *p* < 0.01) comparable to 50% with STP (**, *p* < 0.005). *c*, high magnification of samples analyzed in Fig. 2*a*. Mitochondria (*arrows*) of HeLa cells transfected with ASO-1537S are more electron-dense and show collapse of the matrix compared with the normal morphology of control cells. *Bars* = 500 nm. *d*, quantification analysis of TEM microphotographs indicate that ASK induces ∼82% of cells with collapsed mitochondria. The *numbers on top of each bar* indicate cells with altered mitochondria *versus* total amount of cells analyzed. *e*, dissipation of ΔΨm. HeLa cells were transfected with 100 nm ASO-C or ASO-1537S or left untreated for 24 h. A parallel NT culture was incubated with the uncoupling drug CCCP (see “Experimental Procedures”). Cells were then harvested, stained with 20 nm tetramethylrhodamine methyl ester for 15 min, and analyzed by flow cytometry. ASK with ASO-1537S induces dissipation of ΔΨm comparable to that obtained with CCCP. *TMRM*, tetramethylrhodamine methyl ester fluorescence intensity. *f*, a triplicate analysis shows that ASO-1537S induces ∼50% dissipation of ΔΨm compared with 70% for CCCP. *g*, dissipation of ΔΨm in additional tumor cell lines. DU-145 cells (prostate carcinoma), MDA-MB-231 cells (breast carcinoma), and H292 cells (lung carcinoma) were seeded at 5 × 10^4^ cells/well. On the next day the cells were transfected with ASO-C or ASO-1537S under the conditions listed in Table 1. At 24 h post-transfection, cells were processed as in *b* and analyzed by flow cytometry. ASK induced considerable dissipation of ΔΨm as compared with ASO-C.

In another approach HeLa cells treated in the same way were fixed, stained with PI, and analyzed by flow cytometry. STP-treated cells were used as positive control. A significant sub-G_1_ fraction, indicative of DNA fragmentation, was detected only in HeLa cells treated with ASO-1537S or STP but not in control cells (Fig. 3, *a* and *b*).

##### Cell Death Elicited by ASK Displays Features of Apoptosis

High magnification TEM of HeLa cells transfected with ASO-1537S for 48 h reveals alteration of mitochondrial morphology and matrix collapse compared with controls (Fig. 3*c*). A quantification analysis of TEM records indicates that ASK induces ∼80% of cells with collapsed mitochondria (Fig. 3*d*). Because there is a close correlation between mitochondrial morphological alterations and disruption of the mitochondrial membrane potential (ΔΨm) (27, 30–32), we determined whether ASK affects ΔΨm. HeLa cells treated as above were harvested and incubated with the fluorescent probe tetramethylrhodamine methyl ester. The uncoupling drug CCCP was used as a positive control (27). Flow cytometry revealed that, as with CCCP, treatment with ASO-1537S caused a marked dissipation of ΔΨm, compared with controls (Fig. 3*e*). The results of three independent experiments show that ASO-1537S and CCCP induce ∼55 and 70% dissipation of ΔΨm, respectively, compared with 10–15% in controls (Fig. 3*f*). ASK also induces ΔΨm dissipation in DU145 (prostate carcinoma), MDA-MB-231 (breast carcinoma), and H292 cells (lung carcinoma) (Fig. 3*g*).

**FIGURE 4.**
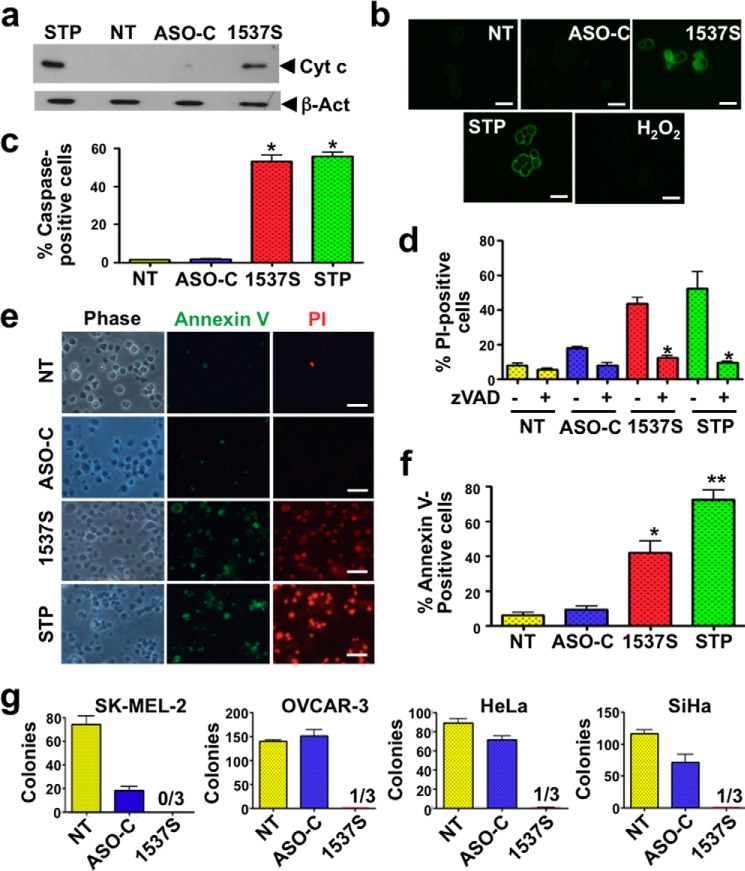
**Tumor cell death elicited by ASK displays hallmark features of apoptosis.** *a*, release of cytochrome *c* (*Cyt c*)from mitochondria. HeLa cells were transfected as described in *b* or incubated with 5 μm STP. At 24 h, Western blot of the cytosolic fraction (see “Experimental Procedures”) reveals that cytochrome *c* was present in the cytosolic fraction only of cells transfected with ASO-1537S or incubated with STP. β*-Act*, β-actin. *b*, ASK induces caspase activation. HeLa cells were transfected in triplicate with 100 nm ASO-C or ASO-1537S for 24 h and labeled with FITC-VAD-fmk (see “Experimental Procedures”). Parallel cultures were incubated with 5 μm STP to induce apoptosis or 100 μm H_2_O_2_ to induce necrosis or left untreated (*NT*). Only cells treated with ASO-1537S or STP exhibited green fluorescence. The images correspond to green fluorescence plus phase contrast. *Bars* = 10 μm. *c*, treatment with ASO-1537S or STP induced a significant fraction of FITC-VAD-fmk-positive cells, compared with controls (*, *p* < 0.01). *d*, inhibition of cell death by z-VAD-fmk. HeLa cells were transfected with ASO-C, ASO-1537S, left untreated (NT), or incubated with STP as in *b* and with or without 25 μm z-VAD-fmk for 24 h. Viability was determined with PI staining and flow cytometry. A triplicate assay shows that z-VAD-fmk significantly inhibited cell death induced by ASK or STP (*, *p* < 0.01). *e*, ASK induces annexin V-positive cells. HeLa cells were treated for 24 h with ASO-1537S, ASO-C, or STP or left untreated (*NT*), labeled with annexin V-Alexa Fluor 488, and stained with PI. As with STP, ASO-1537S induced annexin-positive cells. Controls (*NT* and *ASO-C*) displayed very few annexin V and PI-labeled cells. *Bars* = 50 μm. *f*, a triplicate analysis revealed that ASK or STP induce a significant fraction of 40% (*, *p* < 0.01) and 70% (**, *p* < 0.005) of annexin V-positive cells, respectively, as compared with <10% in controls (NT and ASO-C). *g*, inhibition of anchorage-independent growth. SK-MEL-2, OVCAR-3, HeLa, and SiHa cells were transfected for 48 h with ASO-C or ASO-1537S under the conditions described in Table 1. After harvesting and counting, 200 cells (HeLa and SiHa), 500 cells (OVCAR-3), or 2000 cells (SK-MEL-2) were seeded in soft agar (see “Experimental Procedures”). Cultures were carried out in triplicate, and colonies >50 μm in diameter were scored at 2–3 weeks. Numbers over the 1537S *bars* represent total number of colonies in 3 wells.

Because dissipation of ΔΨm induces release of cytochrome *c* from mitochondria followed by activation of caspases (27, 30–32), HeLa cells were incubated with STP or transfected for 24 h with ASO-1537S or ASO-C or left untreated (NT). Cells were harvested and processed to obtain the cytosolic fraction (see “Experimental Procedures”). Western blot analysis revealed the presence of cytochrome *c* in the cytosolic fraction, only from cells treated with STP or ASO-1537S and not from control cells (Fig. 4*a*).

To determine whether ASK provokes activation of caspases, HeLa cells were transfected for 24 h as described or treated with STP or with 100 μm H_2_O_2_ as the necrosis control. Cells were then incubated with the fluorescent pan inhibitor of caspases FITC-VAD-fmk, which binds to activated caspases (33) (see “Experimental Procedures”). Fluorescence was found only in cells treated with ASO-1537S or STP (Fig. 4*b*). A triplicate analysis at 24 h revealed that ∼50% of cells treated with ASO-1537S or STP contained activated caspases (Fig. 4*c*). To confirm that caspase activation was involved in cell death, we determined whether the non-fluorescent z-VAD-fmk would inhibit ASK-induced cell death. HeLa cells were treated as above with or without 25 μm z-VAD-fmk and then stained with PI. Total treatment time in this experiment was 48 h as plasma membrane permeabilization that will allow free entrance of PI is a late event in apoptosis that occurs much after caspase activation. The proportion of PI-positive *versus* total cells indicated that cell death induced by ASO-1537S or STP was markedly inhibited by z-VAD-fmk (Fig. 4*d*).

To measure translocation of phosphatidylserine to the outer layer of the plasma membrane, HeLa cells treated as above were incubated with annexin V-Alexa Fluor 488 and counterstained with PI. A representative result shows that HeLa cells treated with ASO-1537S were positive for annexin V and PI, similarly to cells treated with STP (Fig. 4*e*). At 24 h post-transfection, ASK yields ∼50% annexin V-positive cells compared with 75% of cells treated with STP (Fig. 4*f*).

##### Inhibition of Anchorage-independent Growth

A noteworthy property of transformed cells is the capacity of anchorage-independent growth, and thus colony formation in soft agar is considered a parameter of tumorogenicity (19). We determined whether ASK affects this property. SK-MEL-2, OVCAR-3, HeLa, and SiHa cells were transfected with ASO-1537S or ASO-C (Table 1) or left untreated (NT). Cells were harvested, counted, and seeded in 12-well plates on soft agar as described under “Experimental Procedures.” Depending on the cell line, between 200 and 2000 live cells, as determined by Tb exclusion, were seeded per well. After 2–3 weeks in culture, colonies >50 μm in diameter were counted. ASK induces a drastic inhibition of colony formation in all tumor cell lines assayed (Fig. 4*g*).

##### Viability of Normal Cells

**FIGURE 5.**
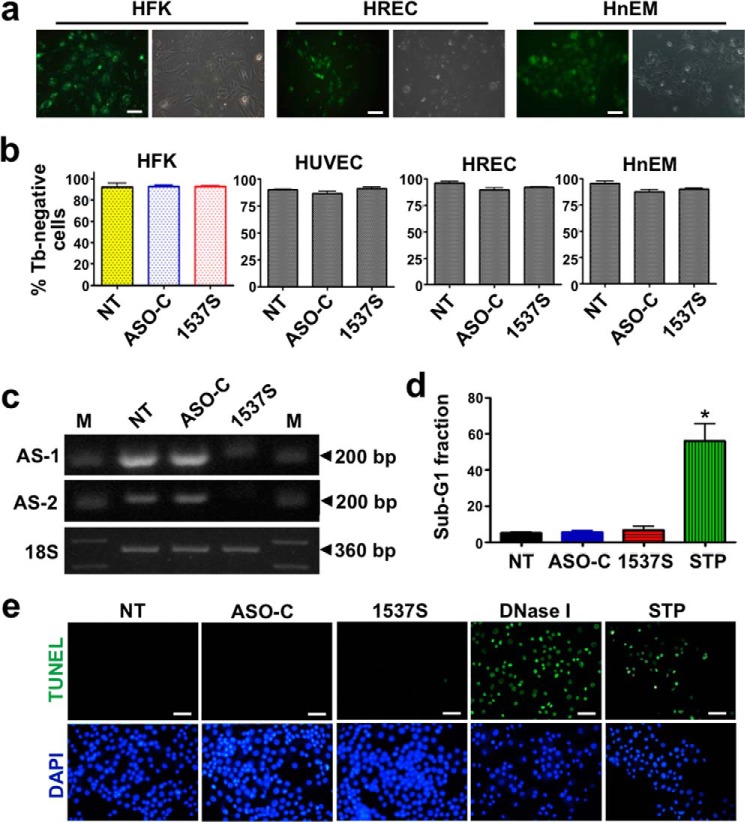
**ASK does not affect viability of normal cells.** *a*, HFK, HREC, and HnEMs were seeded at 5 × 10^4^ cells per well in 12-well plates. On the next day the cells were transfected with ASO-1537S conjugated to Alexa Fluor 488 under the conditions listed in Table 1. At 24 h post-transfection at least 90% of the cells were fluorescently labeled (fluorescence, *left panel*; phase contrast, *right panel* in each case) (*bars* = 20 μm). *b*, HFK (*colored graph*), HUVEC, HREC, and HnEMs were seeded at 5 × 10^4^ cells/well in 12-well plates and transfected the next day with ASO-C or ASO-1537S (see Table 1) or left untreated *(NT*). Cells treated for 48 h were counted by Tb exclusion. A triplicate analysis showed that viability of these cells was unaffected by ASK. *c*, HFK transfected as in *b* for 48 h were harvested, counted, and subjected to RNA extraction followed by RT-PCR amplification. A representative gel shows that ASO-1537S induces a decrease in levels of AS-1 and AS-2. The 18 S rRNA was used as the loading control (*M*, 100-bp ladder). *d*, same as in *b*, but harvested cells were fixed, stained with PI and analyzed by Flow cytometry. STP was used as a positive cell death control. A triplicate analysis revealed that only STP induced a significant sub-G_1_ DNA fraction (60%) in HFKs (*, *p* < 0.01), whereas the sub-G_1_ fraction induced by ASK was similar to that of ASO-C and NT. *e*, ASK does not induce TUNEL-positive cells in HFK. HFK were seeded and transfected as in *b*. After 24 h cells were labeled with fluorescent TUNEL assay, counterstained with DAPI, and analyzed under fluorescence microscopy. DNase I treatment was carried out as a positive control for TUNEL (*bars* = 20 μm).

Next we asked whether ASK also affects the viability of normal human cells in culture. HFK, primary HREC, and melanocytes (HnEM) displayed >90% transfection at 24 h with Alexa Fluor 488-ASO-1537S (Fig. 5*a*). However, viability of HFK, HUVEC, HREC, and HnEM evaluated by Tb exclusion assay was not affected by ASK (Fig. 5*b*). Moreover, ASK in HFK with ASO-1537S for 48 h induced knockdown of ASncmtRNA-1 and ASncmtRNA-2 (Fig. 5*c*). On the other hand, a considerable sub-G_1_ fraction in HFK was only observed with STP treatment but not in cells transfected with ASO-C, ASO-1537S, or untreated (Fig. 5*d*). This result was confirmed by TUNEL assay. TUNEL-positive HFKs were observed only after treatment with STP and DNase I (Fig. 5*e*).

##### ASK Induces Down-regulation of Survivin

**FIGURE 6.**
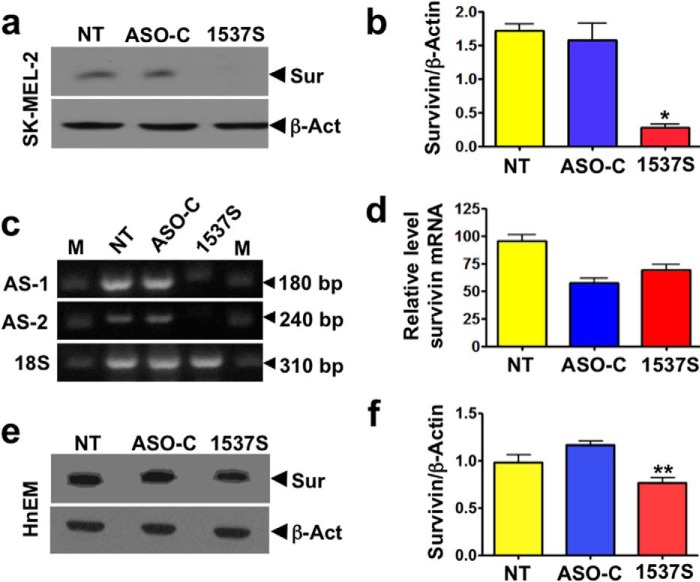
**ASK induces down-regulation of survivin.** *a*, SK-MEL-2 cells were seeded in 6-well plates at 10^5^ cells/well and transfected the next day with 150 nm ASO-C or ASO-1537S under the conditions described in Table 1 or left untreated (*NT*). At 24 h, cells were processed for Western blot (see “Experimental Procedures”). Survivin (*Sur*) was drastically decreased by ASK compared with controls (NT and ASO-C). β*-Act*, β-actin. *b*, a triplicate densitometric analysis of the experiment in *a* for SK-MEL-2 cells indicated that ASK induced ∼80% decrease in survivin compared with controls (*, *p* < 0.01). *c*, RT-PCR revealed that ASK induces knockdown of both ASncmtRNAs (AS-1 and AS-2) in SK-MEL-2 cells. Amplification of 18 S rRNA was used as the loading control. *d*, RNA from *c* was used to determine the relative expression of survivin mRNA by quantitative real-time-PCR, using 18 S rRNA and RPL27 mRNA as reference genes. A triplicate analysis indicated that the relative expression of survivin mRNA in cells transfected with ASO-1537S was similar to control ASO-C. *e*, survivin expression in melanocytes (HnEMs) as the normal counterpart of melanoma SK-MEL-2 cells. HnEMs were transfected for 48 h as described before with 150 nm ASO-C or ASO-1537S or left untreated. The cell lysate was analyzed with anti-survivin using β-actin as loading control. *f*, a triplicate analysis revealed that the expression of survivin post-ASO-1537S treatment was 70% of that obtained with ASO-C (**, *p* < 0.005). Compared with untreated cells, the difference of expression of survivin was not significant.

The previous results indicate that ASK induces tumor cell death by apoptosis, which depends on the counterbalance between pro- and anti-apoptotic factors (34, 35). Among anti-apoptotic factors, the IAP family plays an important cytoprotective function in cancer cells downstream of the intrinsic apoptosis pathway (15–18). Therefore, we asked whether the expression of survivin, a member of the IAP family, could be affected by ASK. In this study we used the melanoma cell line SK-MEL-2, transfected with 150 nm ASO-C or ASO 1537S for 24 h (Table 1). Cells were then harvested, lysed, and subjected to Western blot using β-actin as the loading control. Transfection with ASO-1537S induced a marked inhibition of survivin expression compared with controls (Fig. 6*a*, *NT* and *ASO-C*). Densitometric analysis of blots from three independent experiments indicated that levels of survivin decreased by ∼80% at 24 h post-transfection of ASO-1537S (Fig. 6*b*). Knockdown of ASncmtRNA-1 and -2 in SK-MEL-2 cells was confirmed by RT-PCR (Fig. 6*c*). In contrast, the relative levels of survivin mRNA in cells treated with ASO-1537S were comparable to those treated with the ASO-C (Fig. 6*d*), suggesting that the decrease in survivin protein was not due to mRNA degradation and could occur at the translational or post-translational level. Because the viability of normal cells is not affected by ASK, we asked whether survivin is down-regulated in this scenario. We used melanocytes (HnEMs), which are the normal counterpart of melanoma SK-MEL-2 cells. HnEMs were transfected as described before, with 150 nm ASO-C or ASO-1537S or left untreated for 48 h. Cells were then harvested, lysed, and subjected to Western blot using β-actin as loading control. A triplicate analysis revealed that the expression of survivin was reduced by ∼30% compared with transfection with ASO-C (*p* < 0.005) (Fig. 6*f*). No significant difference was found compared with the expression of survivin in untreated melanocytes (Fig. 6*f*).

**FIGURE 7.**
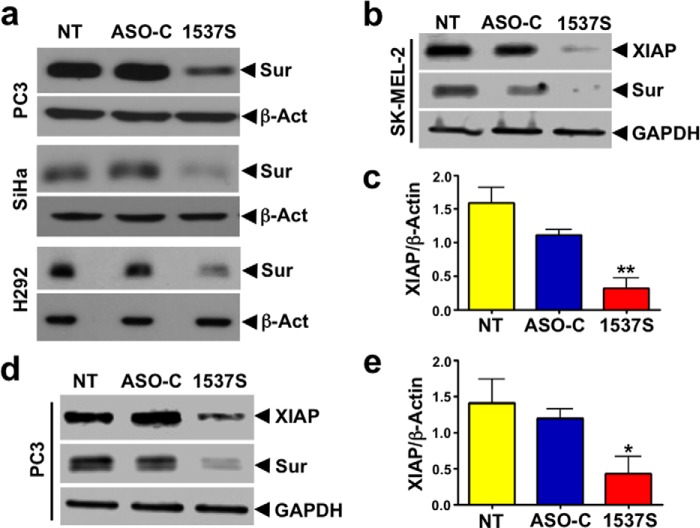
**ASK induces down-regulation of survivin (*Sur*) and XIAP in other tumor cell lines.** *a*, PC3, SiHa, and H292 cells were transfected according to the conditions described in Table 1, with ASO-C or ASO-1537S or left untreated (*NT*), and cell lysates were analyzed by Western blot. Transfection with ASO-1537S also induces down-regulation of survivin in these cells, as compared with controls. β*-Act*, β-actin. *b*, ASK induces down-regulation of XIAP. SK-MEL-2 cells were transfected as described before for 24 h and analyzed by Western blot with anti-XIAP and anti-survivin antibody using GAPDH as loading controls. XIAP and survivin are reduced by ASK, compared with controls. *c*, this result was confirmed in three different experiments (**, *p* < 0.01). *d* and *e*, the same result was obtained with PC3 cells (*, *p* < 0.05).

ASK also induced survivin down-regulation in PC3, SiHa, and H292 cells (Fig. 7*a*). Down-regulation of survivin should affect the expression of XIAP, another member of the IAP family (15, 36, 37). Western blot of SK-MEL-2 cells after transfection with ASO-1537S shows down-regulation of XIAP and survivin (Fig. 7*b*). A triplicate analysis of these blots confirmed down-regulation of XIAP (Fig. 7*c*). Similar results were obtained with PC3 cells (Fig. 7, *d* and *e*).

**FIGURE 8.**
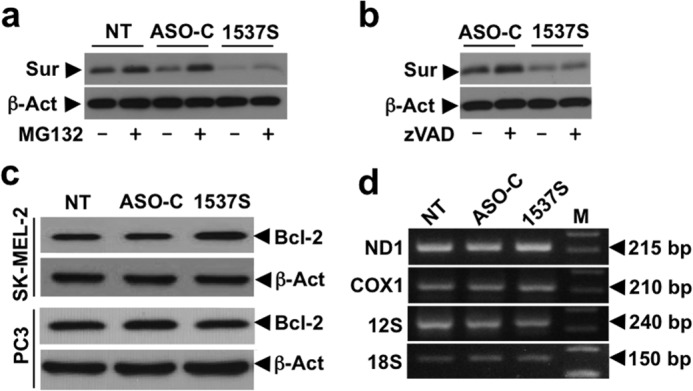
**Down-regulation of survivin (*Sur*) is not affected by proteasome or caspase degradation.** *a*, SK-MEL-2 cells were treated as described in the presence or absence of 5 μm proteasome inhibitor MG132, added 4 h before transfection. Western blot at 24 h post-transfection shows that the inhibitor did not revert the decrease in survivin. β*-Act*, β-actin. *b*, the same as in *a*, but transfection was carried out in the presence or absence of 40 μm z-VAD-fmk for 24 h. The presence of z-VAD-fmk did not significantly revert the reduction in survivin elicited by transfection with ASO-1537S. *c*, ASK does not affect the expression of Bcl-2. SK-MEL-2 and PC3 cells were seeded and transfected as in *a* for 24 h and analyzed by Western blot, revealing that Bcl-2 expression was not affected by ASO-1537S. *d*, ASK does not affect mitochondrial transcription. SK-MEL-2 cells were transfected for 24 h as described before. Total RNA was isolated and subjected to RT-PCR amplification for 25 cycles (ND1 and COX1) or 20 cycles (12 S) and using 18 S rRNA (15 cycles) as control. No change in the expression of ND1, COX1, and 12 S mitochondrial rRNA was observed. *M*, 100-bp ladder.

To determine whether survivin down-regulation was due to proteasomal degradation, SK-MEL-2 cells were transfected for 24 h as before without or with the 26 S proteasome inhibitor MG132 (38). MG132 failed to recover the basal survivin expression in ASO-1537S-treated cells (Fig. 8*a*). To explore the possibility that survivin was degraded by activated caspases (39), ASK in SK-MEL-2 cells was carried out without or with z-VAD-fmk for 24 h. The caspase inhibitor did not revert survivin decrease (Fig. 8*b*). Interestingly, in SK-MEL-2 and PC3 cells, the expression of Bcl-2, another anti-apoptotic factor (40), was not affected by ASK (Fig. 8*c*). Next, we asked whether ASK affects mitochondrial transcription. SK-MEL-2 cells were transfected for 24 h as described before, and total RNA was amplified by RT-PCR. Levels of ND1, COX1, and 12 S mitochondrial rRNA were unaffected by ASK, as was the case for 18 S rRNA used as control (Fig. 8*d*).

##### Cell Death and Down-regulation of Survivin Is RNase H-dependent

**FIGURE 9.**
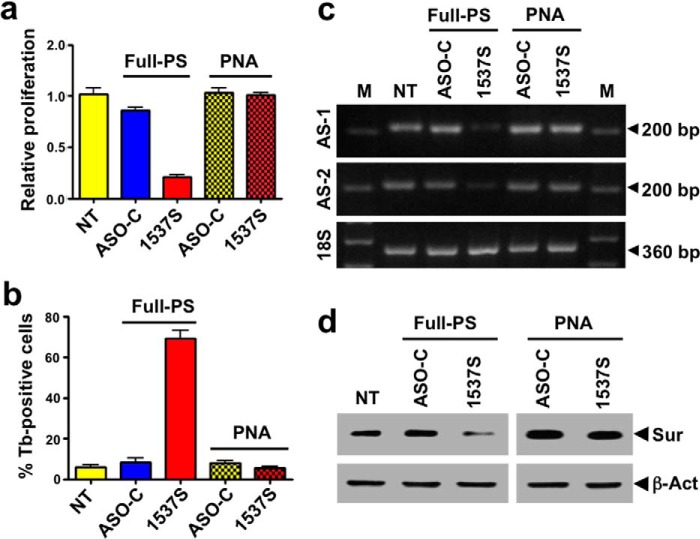
**Cell death and survivin down-regulation is RNase H-dependent.** *a*, SK-MEL-2 cells were transfected with full-phosphorothioate (full-PS) ASO-1537S or PNA-1537S. At 24 h post-transfection, cells were harvested and counted. Only full-PS ASO-1537S elicited inhibition of cell proliferation. *b*, cells were transfected as in *a* for 24 h, after which they were harvested and stained with Tb. A high proportion of Tb-positive cells was only observed in cells transfected with ASO-1537S. *c*, PNA-1537S does not induce knockdown of ASncmtRNAs. Cells were transfected with ASO-1537S or PNA-1537S as in *a* for 24 h followed by RNA extraction and RT-PCR amplification of both ASncmtRNAs. Only ASO-1537S full-PS induced knockdown of the ASncmtRNA-1 and ASncmtRNA-2. *M*, 100-bp ladder. *d*, survivin (*Sur*) down-regulation. SK-MEL-2 cells were transfected as in *a* with ASO-1537S or PNA-1537S, and whole-cell extracts were subjected to Western blot. Down-regulation of survivin was induced only by full-PS ASO-1537S transfection. β*-Act*, β-actin.

The mechanism by which antisense deoxyoligonucleotides with phosphorothioate linkages exert their effects on gene expression is through the formation of ASO·RNA hybrids that are recognized and cleaved by RNase H (41, 42). Transfection of cells with ASO-1537S should result in cleavage of the loop regions of ASncmtRNA-1 and -2 (Fig. 1*b*). Therefore, we asked whether cell death by ASK depends on RNase H cleavage or is just a simple steric hindrance effect of the ASO·RNA hybrid. To test this hypothesis, transfection of SK-MEL-2 cells was carried out with a PNA (42) ASO-1537S. PNA oligonucleotides also form PNA·RNA hybrids, interfering with RNA functions. However, because of the peptidic nature of the PNA backbone, the mechanism of action of PNA is independent of RNase H (42). In contrast to phosphorothioate-ASO-1537S, transfection with PNA-1537S failed to affect proliferation of SK-MEL-2 cells (Fig. 9*a*), to induce cell death evaluated by Tb-exclusion (Fig. 9*b*), or induce knock-down of the ASncmtRNAs (Fig. 9*c*). Moreover, survivin levels were not affected by transfection with PNA-1537S (Fig. 9*d*).

##### Dicer Is Bound to the ASncmtRNAs

**FIGURE 10.**
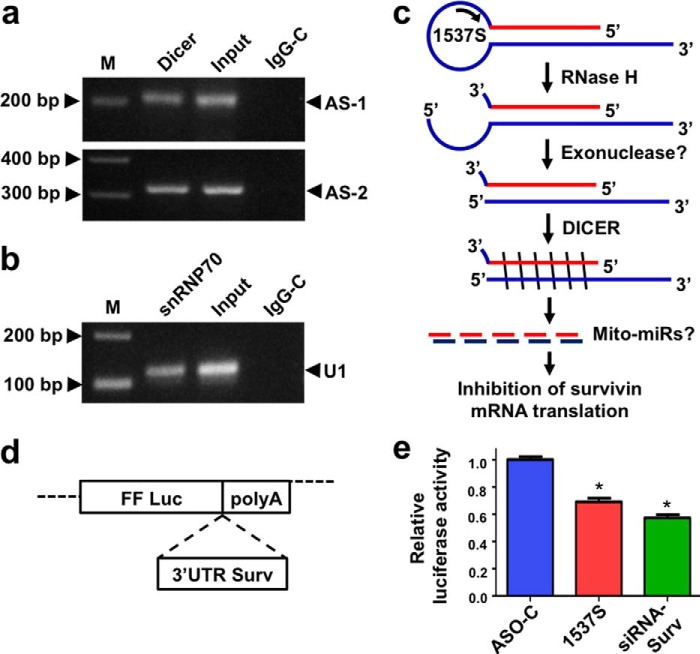
**The ASncmtRNAs are bound to Dicer *in vivo*.** *a*, whole-cell lysates from SK-MEL-2 cells were immunoprecipitated with an anti-Dicer antibody, and RT-PCR amplification of the ASncmtRNAs was performed on total RNA extracted from the IPs. AS-1 and AS-2 were readily amplified from the anti-Dicer IP but not with control IgG. *b*, anti-SNRNP70 was used as a positive control for immunoprecipitation followed by RT-PCR amplification of U1snRNA (*U1*). *c*, schematic representation of ASO-1537S forming a hybrid with the ASncmtRNA-2 loop region followed by cleavage of the hybrid by RNase H. The remainder of the loop is removed, probably by a 5′ to 3′ exonuclease(s), leaving a 3′-OH overhang. The resulting double-stranded transcript is processed by Dicer, generating fragments of ∼22 bp (Mito-miRs?). These putative miRs would interact with 3′-UTR of survivin mRNA inhibiting its translation. ASK induces miR(s) that interact with 3′-UTR of survivin mRNA. *d*, schematic representation of the pmiRGLO dual-luciferase vector carrying the 3′-UTR region of survivin mRNA (3′-UTR survivin) downstream from the firefly luciferase ORF (FF Luc) and just before the SV40 polyadenylation signal (poly(A)). *e*, SK-MEL-2 cells were transfected with the vector containing the 3′-UTR region or empty vector, using Lipofectamine2000. After 24 h cells were transfected with 150 nm ASO-C or ASO-1537S or with 20 nm survivin siRNA used as the positive control. On the next day, the relative luciferase activity was determined (see “Experimental Procedures”). Results are expressed as the ratio of firefly/Renilla luciferase activity, normalized to that of the empty vector (*, *p* < 0.01 respect to cells transfected with ASO-C). *M*, 100-bp ladder.

The above results indicate that induction of cell death and down-regulation of survivin requires RNase H cleavage of the ASO-1537S·ASncmtRNA hybrid at the loop of these transcripts, resulting in the generation of double-stranded transcripts with long 3′ end single-stranded components (see Fig. 10*c*). This scenario opens an important question: Do the resulting transcripts with double-stranded regions constitute substrates for Dicer that could give rise to endo-siRNAs or miR precursors? If so, Dicer should be found associated to the ASncmtRNAs *in vivo*. To address this issue, whole-cell lysates of SK-MEL-2 cells were immunoprecipitated with a Dicer-specific monoclonal antibody followed by RNA extraction from the immunoprecipitation and RT-PCR amplification of the ASncmtRNAs (see “Experimental Procedures”). A representative result (out of five) of RT-PCR amplification confirms the presence of the AS-1 and AS-2 in the immunoprecipitates (Fig. 10*a*). These transcripts were not amplified from IPs with control IgG (Fig. 10*a*, *IgG-C*). U1snRNA (U1) was RT-PCR-amplified from anti-SNRNP70 IPs as control of IP efficiency and specificity (Fig. 10*b*).

##### The Stem of ASncmtRNAs as a Putative Source of MicroRNAs Regulating Survivin Expression

As shown in Fig. 6*d*, ASK did not significantly affect the relative expression of survivin mRNA, suggesting that decrease of survivin protein expression occurs at the translational level and perhaps mediated by miRs. It has been shown that several miRs are involved in regulation of survivin expression (43, 44). We also show that in whole-cell lysates Dicer is bound to the ASncmtRNA-1 and ASncmtRNA-2 (Fig. 10*a*). ASK with ASO-1537S will induce cleavage of the loop by RNase H, generating a double-stranded structure or stem in the transcripts (Fig. 10*c*). RNase H activity cleaves the 3′-O-P bond of ASncmtRNAs in the ASO·ASncmtRNA duplex to produce 3′-hydroxyl and 5′-phosphate-ended products. Next, a 5′-3′ exonuclease will probably eliminate the 5′ overhang segment derived from the loop, leaving a 5′-phosphate terminus. This stem displays characteristics for 3′ or 5′ counting models to generate ∼22-bp fragments (Fig. 10*c*) (24). Hypothetically then, it is possible that the long stem will be processed by Dicer, generating double-stranded fragments of ∼22 bp. Indeed, *in silico* dicing of the ASncmtRNA-2 stem, for example, generated several hypothetical ∼22-bp fragments (20, 28, 29, 45), containing at the 5′ end the seed region that could interact with the miR recognition element (20, 28, 29, 45) located at the 3′-UTR of survivin mRNA. To test the hypothesis that ASK induces miRs, which are involved in survivin down-regulation, we used a luciferase reporter vector encoding the survivin 3′-UTR region downstream of the firefly luciferase gene (Fig. 10*d*). The empty vector and a siRNA targeted to the 3′-UTR of survivin were used as controls. As shown in Fig. 10*e*, the relative activity of firefly to Renilla luciferase in SK-MEL-2 cells transfected with pmiRGLO-survivin 3′-UTR and ASO-1537S was ∼70% (six independent experiments) of control cells transfected with pmirGLO-survivin 3′-UTR and ASO-C. Similarly, the ratio of cells transfected with the survivin siRNA was ∼60% of the control. Taken together, these results strongly suggest that ASK induces the generation of miRs (Mito-miRs?) that interact with the 3′-UTR of survivin mRNA, consequentially inhibiting luciferase expression.

## DISCUSSION

Hallmarks of cancer are fundamental principles involved in malignant transformation (1). In harmony with these principles, a large number of key proteins involved in these processes have been identified (1). Small ncRNAs (*e.g.* microRNAs) (46, 47) and long ncRNAs (48) also play important roles in the modulation of each of these properties. In this context we reported that mitochondrial long ncRNAs (SncmtRNA and the ASncmtRNAs) are also related to neoplastic transformation, and here we propose that the generalized down-regulation of the ASncmtRNAs in cancer cells is a novel pro-tumorigenic hallmark of cancer. One hallmark of cancer is down-regulation or abolishment of the function of tumor suppressors involving several mechanisms including proteins from oncogenic viruses such as HPV (9, 10). For example, oncoproteins E6 and E7 from high risk HPV 16 and 18 abolish the function of two tumor suppressors: p53 and Rb (10). Because in HeLa and SiHa cells transformed with HPV 18 or 16, respectively, the ASncmtRNAs are also down-regulated, we hypothesized that these transcripts were mitochondria-encoded tumor suppressors (6). To test this hypothesis we studied human keratinocytes immortalized with HPV 16 and 18. We determined that the oncoprotein E2 of these viruses is involved in down-regulation of ASncmtRNAs, supporting our hypothesis that the ASncmtRNAs are tumor suppressors (8).

However, a very intriguing question remains: Why are the ASncmtRNAs not suppressed completely in cancer cells? We have speculated that perhaps the low copy number of ASncmtRNAs may also be beneficial to cancer progression but now function as a pro-survival factor. In other words we propose that in some situations a tumor suppressor is converted into a pro-survival factor or proto-oncogene. Supporting this speculation is the property of a missense mutation of p53 (mp53) existing in >50% of human cancers. Recently the conjecture is that the mutated protein has acquired, besides its effect on wild-type p53, a novel function or gain-of-function that contributes to cancer progression and metastasis. Moreover, silencing mp53 with RNAi induces massive apoptosis and diminishes the metastatic potential of cancer cells (49–54). Similarly, knockdown of the few copies of the ASncmtRNAs induces massive cancer cell death by apoptosis and drastic inhibition of colony formation. Nevertheless, this issue warrants future studies.

However, the low copy number of the ASncmtRNAs also seems to be a vulnerability of cancer cells. Indeed, knocking down (ASK) these transcripts is a potent and selective means to induce cell death in all cancer cell lines tested. Here we show that ASK induces dissipation of the ΔΨm followed by release of cytochrome *c*, activation of caspases, DNA fragmentation, and other classical hallmarks of apoptosis (14). Interestingly, however, the viability of normal cells is unaffected by the same treatment (Fig. 5). In addition, ASK induces marked inhibition of anchorage-independent growth of SK-MEL-2, OVCAR-3, SiHa, and HeLa cells (Fig. 4). Anchorage-independent growth correlates closely to tumorigenic capacity in transformed cells (17), suggesting a potential use of the ASncmtRNAs as a target for cancer therapy *in vivo*.

The onset of apoptotic cell death is potentiated by down-regulation of survivin, an important anti-apoptotic factor. Survivin is a member of the IAP family, which is up-regulated in virtually all human cancers (15–18). This generalized feature has inspired multiple efforts in using survivin as a novel target for cancer therapy (55). As shown here, ASK induces a drastic reduction in intracellular levels of survivin in SK-MEL-2, PC3, SiHa, and H292 cells. Interestingly, XIAP, another member of the IAP family of proteins, was also down-regulated by ASK in SK-MEL-2 and PC3 cells. This is a rather expected result as one function of survivin is to form a complex with XIAP to stabilize the inhibitory effect on caspases (15, 36, 37).

Although degradation of survivin induced by ASK in the proteasome or by activated caspases seems unlikely, we cannot discard other routes of degradation. On the other hand, ASK did not significantly affect the relative expression of survivin mRNA; taken together, these results suggest that inhibition of survivin expression occurs at the translational level and is probably mediated by miRs (20–24). Several miRs that regulate survivin expression have been described (43, 44).

Which is the source of these putative miRs induced by ASK? It is important to mention that there is no sequence within the ASncmtRNAs that is fully complementary as a siRNA, to target survivin mRNA. One possibility is that ASK induces the generation of miRs, probably from the stem of the ASncmtRNAs. An intriguing question is how these mitochondrial transcripts containing stem-loop structures escape from the processing activity of Dicer. The double strands of the ASncmtRNAs should bind to the double-stranded binding domain of Dicer (56, 57). Here we show for the first time that in whole-cell lysates a fraction of Dicer molecules forms complexes with the ASncmtRNAs. However, to validate these results we must carry out EMSA experiments. These experiments require templates for synthesis of the ASncmtRNAs *in vitro*. Unfortunately, and as reported before (5, 6), cloning of these transcripts has proven unsuccessful. The explanation for this failure is quite simple as the long double-stranded stem represents an insurmountable problem for the DNA polymerases routinely used in cDNA synthesis (5, 6). The poor strand separation activity of these enzymes results in replication slippage on templates carrying hairpin structures (58, 59). In addition, thermophilic DNA polymerases, including Taq polymerase, also exhibit replication slippage on templates containing hairpin structures, with fateful consequences for amplification and DNA sequencing (60, 61). Undoubtedly, EMSA is important and warrants future efforts. At present we are working on the difficult task of constructing synthetic DNA templates containing the complete sequence of these transcripts.

Down-regulation of survivin and XIAP by ASK is not a generalized phenomenon on anti-apoptotic proteins. Besides the expression of β-actin and GAPDH, the expression of Bcl-2 was not affected by ASK. Similarly, the expression of the mitochondrial mRNAs of ND1 and COX1 and the 12 S rRNA was the same as the controls. These results were rather expected as DNA or RNA oligonucleotides are not able to cross the double membrane of mitochondria by conventional techniques similar to the transfection protocol described in this work (62–65).

**FIGURE 11.**
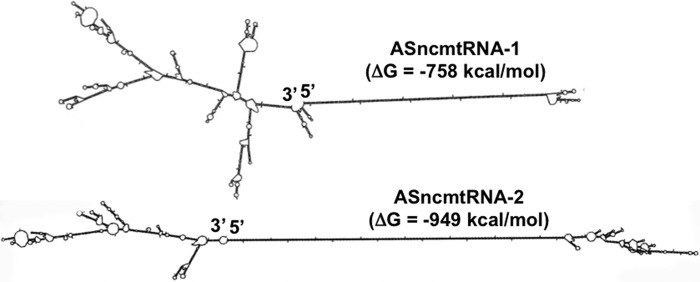
**The theoretical secondary structure of ASncmtRNA-1 and ASncmtRNA-2 obtained with the mfold software (67).** The 5′ and 3′ ends are indicated. Notice that the double-stranded regions of both transcripts are flanked by complex folding of the “loop” and 3′ end single-stranded regions.

Then why is the stem of these transcripts not processed by Dicer *in vivo*? Perhaps, binding of these transcripts inhibits dicing activity. Indeed, both transcripts recovered together with Dicer after IPs were amplified as expected (6). An interesting example of this is the nuclear-encoded ncRNA *rncs-1,* which is up-regulated during starvation in *Caenorhabditis elegans* (66). This transcript contains branched structures at both extremes of a central double-stranded region of ∼300 bp, similarly to the ASncmtRNAs. Indeed, RNA secondary structure prediction (67) reveals complex branched structures at the loop and the 3′ single-stranded region of ASncmtRNA-1 and ASncmtRNA-2 (Fig. 11). *Rncs-1* also inhibits Dicer activity, and the inhibition is relieved if the branched structures are removed (66). Other ncRNAs containing stem-loop structures are also known to inhibit Dicer (68, 69). Thus, the characteristics of the terminal loop in pre-miR-30 for example contribute to modulate Dicer activity (69). ASK-induced cell death and down-regulation of survivin require cleavage of the loop by RNase H at the ASO-1537/ASncmtRNA hybrids. As shown here, PNA-1537S, which is independent of RNase H activity, fails to induce cell death and survivin down-regulation. Similarly to *C. elegans rncs-1*, the lack of the branched structures from the loop of the ASncmtRNA-2, for example, will relieve the inhibition of Dicer, allowing processing of the double-stranded region of the transcript, generating miRs (Mito-miRs). Using bioinformatic tools (20, 28, 29, 45), we processed the double-stranded stem of this transcript *in silico* and found several hypothetical fragments of ∼22 bp that contain putative 5′ seed sequences for interaction with the 3′-UTR of survivin mRNA, which could potentially block its translation. Although we cannot discard other possibilities, the inhibition of luciferase activity of a vector carrying the 3′-UTR of survivin mRNA as a consequence of ASK strongly suggests that knocking down the ASncmtRNAs induces processing of the stem of these transcripts by Dicer generating miR(s) that inhibit survivin expression. Interestingly, other groups have found miRs localized in isolated mitochondria (70–72).

ASK also induces dissipation of ΔΨm, which correlates with release of cytochrome *c* and other mitochondrial proteins from the intermembrane space (30–32, 73). Single-cell studies upon STP challenge show that the kinetics of cytochrome *c* and Smac/DIABLO release were rather similar and take place before the onset of ΔΨm dissipation (73). Hence, dissipation of ΔΨm seems to be a consequence of the events leading to the release of these proteins. Survivin is localized in mitochondria of cancer cells but not in normal cells (15–18). After STP challenge, survivin is released from mitochondria, and down-regulation of survivin induced by ASK might trigger ΔΨm dissipation. The mechanism by which ASK induces dissipation of ΔΨm requires further clarification.

As shown here, ASK did not affect the viability of four normal human cell types. The resistance mechanism of normal cells is unclear. Contrary to the drastic down-regulation of survivin in SK-MEL-2 and other tumor cell lines induced by ASK, this treatment reduced the expression of survivin by only 30% compared with the control ASO-C (Fig. 6*f*). Perhaps this smaller reduction of survivin may explain why ASK does not affect the viability of melanocytes or other normal cells. However, this result is paradoxical since, according to our model (see Fig. 10*c*), miRs should also be generated from the stem of the ASncmtRNAs in normal cells, where ASK induces knockdown of the ASncmtRNAs at similar levels as in cancer cells. This complex matter warrants further research. Nevertheless, these results suggest that down-regulation of the ASncmtRNAs is a vulnerability of cancer cells and ASK is a selective approach to induce cancer cell death without affecting normal cells. Indeed, we have tested ASK *in vivo* using a syngeneic mouse model of melanoma (B16F10 cells). Treatment with an ASO equivalent to ASO-1537S (ASO-1560S), targeted also to the loop of the murine ASncmtRNAs, completely inhibits tumor growth and metastasis of lung and liver. This treatment does not induce inflammatory response or affect body weight of mice. In addition, histopathological analysis of several organs revealed no alteration of healthy tissue of mice treated with ASO-1560S.^5^

## Footnotes

5 L. Lobos-González, V. Silva, S. Vidaurre, J. Echenique, M. Araya, M. Varas, L. Oliveira-Cruz, C. Fitzpatrick, F. Restovic, C. Villota, J. E. Villegas, C. López, T. Socías, L. O. Burzio, and V. A. Burzio, unpublished results.

## Footnotes

4 The abbreviations used are:

ncRNA: non-coding RNA
ncmtRNA: non-coding mitochondrial RNA
SncmtRNA: sense ncmtRNA
ASncmtRNA: antisense ncmtRNA
AS-1: ASncmtRNA-1
AS-2: ASncmtRNA-2
IAP: inhibitor of apoptosis protein
ASK: ASncmtRNA knockdown
ASO: antisense oligodeoxynucleotide
ASO-C: control ASO
miR: microRNA
PI: propidium iodide
PNA: peptide nucleic acid
PS: phosphorothioate
CCCP: carbonyl cyanide 3-chlorophenylhydrazone
ΔΨm: mitochondrial membrane potential
IP: immunoprecipitate
STP: staurosporine
Tb: trypan blue
EdU: 5-ethynyl-2′-deoxyuridine
XIAP: X-linked inhibitor of apoptosis protein
HPV: human papilloma virus
HFK: human foreskin keratinocyte
HnEM: human normal epidermal melanocyte
HREC: human normal renal epithelial cells
HUVEC: human umbilical vein endothelial cells
RT: room temperature
FISH: fluorescence *in situ* hybridization
TEM: transmission electron microscopy
NT: not treated
z-VAD-fmk: benzyloxycarbonyl-Val-Ala-Asp-fluoromethyl ketone.
